# A Rare Case of Myelofibrosis Progressing to BCR-ABL1-Positive Chronic Myeloid Leukemia With Discordant Molecular Testing

**DOI:** 10.7759/cureus.86975

**Published:** 2025-06-29

**Authors:** Himani Badyal, Vallabh Dogra, Ratika Dogra, Abhay Shelke

**Affiliations:** 1 Internal Medicine, St. Vincent's Medical Center, Toledo, USA; 2 Hematology Oncology, St. Vincent's Medical Center, Toledo, USA

**Keywords:** bcr-abl, chronic myeloid leukemia, fish (fluorescence in situ hybridization), myelofibrosis, philadelphia chromosome

## Abstract

Myelofibrosis is a chronic myeloproliferative neoplasm (CMN) characterized by bone marrow fibrosis, splenomegaly, cytopenias, or cytoses, and a propensity for transformation into acute myeloid leukemia (AML). Transformation of myelofibrosis into chronic myeloid leukemia (CML), however, is extremely rare and poorly understood. CML is typically defined by the presence of the BCR-ABL1 fusion gene, a product of the Philadelphia chromosome t(9;22)(q34;q11), which leads to constitutive activation of a tyrosine kinase that drives leukemogenesis. This molecular abnormality is considered pathognomonic for CML and is routinely identified through techniques such as fluorescence in situ hybridization (FISH) and reverse transcriptase-polymerase chain reaction (RT-PCR). While the transformation from myelofibrosis to CML is unusual, this case report illustrates an unusual transformation of myelofibrosis to CML, marked by the emergence of a BCR-ABL1-positive clone detectable by fluorescence in situ hybridization (FISH) but not by polymerase chain reaction (PCR). It underscores the importance of using multiple complementary molecular diagnostic techniques when evaluating disease progression in myeloproliferative neoplasms. Early recognition of such atypical transformations is essential, as it can open the door to targeted therapies that may significantly alter the patient's prognosis and clinical trajectory.

## Introduction

Myeloproliferative neoplasms (MPNs) are a group of uncommon hematologic disorders characterized by the abnormal proliferation of blood cell lineages due to bone marrow dysfunction. Among these, chronic myeloid leukemia (CML) and myelofibrosis represent two distinct clinical entities. CML is typically defined by the presence of the BCR-ABL1 fusion gene, which results from a reciprocal translocation between chromosomes 9 and 22, commonly referred to as the Philadelphia chromosome (t(9;22)(q34;q11)). In contrast, myelofibrosis is a Philadelphia chromosome-negative neoplasm most frequently associated with mutations such as JAK2 V617F, CALR, or MPL.

Although rare, there have been reported cases in which patients initially diagnosed with myelofibrosis later developed CML, coinciding with the emergence of the BCR-ABL1 fusion gene. The diagnosis of CML relies on the detection of this fusion gene, which can be identified through a variety of molecular and cytogenetic techniques, including fluorescence in situ hybridization (FISH), polymerase chain reaction (PCR), and conventional karyotyping.

In most cases of CML, the BCR-ABL1 fusion is detectable by both FISH and PCR assays. However, in rare instances, such as the case presented here, a patient may exhibit a discordant testing profile, with a positive FISH result for BCR-ABL1 and a negative PCR result. This discrepancy may indicate the presence of a newly emerged leukemic clone harboring a cryptic or variant BCR-ABL1 rearrangement that escapes detection by conventional PCR, possibly due to an atypical breakpoint or low transcript levels.

Such cases highlight the importance of employing complementary diagnostic modalities in the evaluation of suspected clonal evolution, particularly in patients with MPNs undergoing atypical disease progression. Early identification of BCR-ABL1-positive clones, even in the absence of confirmatory PCR results, has significant therapeutic implications, as it may warrant initiation of targeted therapy with tyrosine kinase inhibitors (TKIs).

## Case presentation

We report the case of a 65-year-old female patient with a complex medical history including coronary artery disease status post quadruple coronary artery bypass grafting (CABG), multiple coronary stents, ischemic cardiomyopathy with a severely reduced ejection fraction of 20%, type 2 diabetes mellitus, and chronic obstructive pulmonary disease (COPD). She was referred to the hematology/oncology clinic after routine laboratory tests revealed thrombocytopenia.

At her initial evaluation, comprehensive testing was performed, including complete blood count (CBC), iron studies, ferritin, vitamin B12, folate, serum kappa and lambda light chains, protein electrophoresis, haptoglobin, and peripheral blood smear. Her platelet count was low at 68,000/µL. Serology was positive for Epstein-Barr virus (EBV) viral capsid antigen IgM and IgG. The peripheral smear revealed nucleated red blood cells, normocytic anemia, thrombocytopenia, and a leukoerythroblastic picture. Bone marrow biopsy showed hypocellular marrow with features suggestive of myelofibrosis. Molecular testing identified a JAK2 mutation, and an ultrasound confirmed mild splenomegaly. Based on these findings, she was diagnosed with myelofibrosis and started on Ruxolitinib at 5 mg twice daily, with plans to gradually increase the dose to 15 mg and then 20 mg daily (Table 1).

**Table 1:** Serial Complete Blood Count (CBC) Values Over the Clinical Course

Days	Day 1 (A)	8 months after day 1 (B)	12 months after day 1 (C)	16 months after day 1 (D)	20 months after day 1 (E)	24 months after day 1 (F)
WBC (3.5-11.3 k/ul)	6.5	7.9	88.7	9.3	95.8	108
Hemoglobin (13-17 g/dl)	11.7	11.2	9.9	9.4	9.8	8.8
Hematocrit (40.7-50.3%)	37	34.2	30.4	28.7	30.1	28.2
Platelet Count (138-453 k/ul)	68	36	61	73	130	27
MCV (82.6-102.9 fl)	87.4	90	86	87.5	84.4	85.6
Segmented neutrophils (36-65 %)	41	43	67	64	50	71
Lymphocytes (24-43 %)	26	34	16	17	13	2
Monocytes (3-12 %)	9	15	4	18	9	10
Eosinophils (1-4 %)	0	2	0	1	1	0
Basophils (0-2%)	1	2	0	0	2	0

Despite the treatment on weekly CBC, her platelet count remained persistently low, fluctuating between 30,000 and 50,000/µL, and therefore her Ruxolitinib dose was maintained at 5 mg twice daily.

During one of the follow-ups, CBC revealed a marked increase in white blood cell count to 80,000/µL, hemoglobin of 9.9 g/dL, and persistent thrombocytopenia with platelets at 40,000/µL. Sudden leukocytosis raised the concern for leukemic transformation, which prompted emergent evaluation. On further workup, her peripheral smear showed pronounced neutrophilia with a left shift, 1% circulating blasts, mild normocytic anemia, and severe thrombocytopenia. Bone marrow biopsy was consistent with a chronic myeloproliferative neoplasm, with underlying primary myelofibrosis.

Notably, despite the marrow findings, her white blood cell count continued to rise. Serial reverse transcriptase PCR tests for BCR-ABL1 were negative, but due to the high clinical suspicion, she was evaluated with flow cytometry and FISH testing. She tested positive for BCR-ABL1 on FISH and negative on conventional RT-PCR. This was suggestive of a new chronic myeloid leukemia (CML) clone.

Given these findings, treatment options included hydroxyurea or imatinib. However, frequent hospitalizations for congestive heart failure (CHF) and COPD exacerbations led to a delay in treatment initiation. The patient was eventually started on hydroxyurea, which modestly controlled her white blood cell count, with plans to transition to imatinib as an outpatient.

Imatinib was finally started with improvement in the blood counts, but she experienced persistent nausea and vomiting, leading to the discontinuation of the drug by the patient. Considering her cardiovascular risks, alternative tyrosine kinase inhibitors such as Nilotinib and Dasatinib were deemed unsuitable, and the decision was made to monitor blood counts every four weeks after stopping imatinib.

Unfortunately, her white blood cell count increased again within months, prompting re-initiation of imatinib at a reduced frequency alongside antiemetics. Persistent gastrointestinal side effects led to the cessation of imatinib, and the decision was made to switch to Nilotinib with Hydroxyurea as a bridge. The patient experienced multiple hospitalizations for CHF and COPD exacerbations, complicating the treatment.

Ultimately, progressive multiorgan failure and worsening leukemic transformation led the family to choose comfort care measures. The patient subsequently passed away.

## Discussion

Myeloproliferative neoplasms (MPNs) are clonal hematopoietic stem cell disorders characterized by bone marrow hyperplasia and elevated peripheral blood counts [1]. The major subtypes of MPNs include chronic myeloid leukemia (CML), primary myelofibrosis (PMF), polycythemia vera, and essential thrombocythemia. While CML is defined by the presence of the BCR::ABL1 fusion gene, PMF typically involves mutations such as JAK2, representing two genetically distinct entities within the MPN spectrum.

Primary myelofibrosis, a Philadelphia chromosome-negative MPN, presents with hypercellular bone marrow featuring abnormal, hypolobulated megakaryocytes and progressive fibrosis, often accompanied by peripheral cytopenias or cytoses [2]. The exact mechanisms underlying marrow fibrosis remain incompletely understood. However, somatic mutations affecting hematopoietic stem cells, particularly JAK2, found in approximately 50% of PMF patients, play a significant role. These mutations activate the JAK/STAT pathway, promoting the aberrant expression of fibrogenic cytokines like transforming growth factor-beta, which drive fibrosis.

Clinically, patients with myelofibrosis often exhibit anemia, teardrop-shaped erythrocytes on peripheral smears, and “dry tap” bone marrow aspirations due to fibrosis. The disease course is highly variable, with a median survival of around six years. Early mortality, as in the present case, is commonly due to complications such as bleeding, infections, or marrow failure [3].

In rare cases, patients with primary myelofibrosis may undergo leukemic transformation involving the emergence of a BCR::ABL1-positive clone [4]. This phenomenon reflects the dynamic clonal evolution seen in MPNs, where additional mutations or cytogenetic abnormalities contribute to disease progression. Such a transformation from PMF to CML is extremely uncommon but has been documented.

In patients with known myelofibrosis, the development of atypical hematologic findings, such as persistent leukocytosis, should prompt evaluation for possible clonal evolution or transformation. Myelofibrosis typically presents with anemia, leukopenia or leukocytosis, teardrop cells, and fibrotic marrow with dry tap aspirations. In contrast, CML is characterized by marked leukocytosis, a marrow dominated by immature granulocytes (blasts), and the presence of the Philadelphia chromosome.

CML is a well-defined MPN driven by the BCR::ABL1 fusion gene, which results from a translocation between chromosome 9 (ABL1) and chromosome 22 (BCR), creating a constitutively active tyrosine kinase that drives uncontrolled myeloid proliferation [5]. This fusion can be detected by various methods, including conventional karyotyping, fluorescence in situ hybridization (FISH), polymerase chain reaction (PCR), and bone marrow analysis. Among these, PCR and FISH are the most sensitive techniques for identifying the BCR::ABL1 fusion [6].

PCR assays quantify BCR::ABL1 transcripts in peripheral blood or bone marrow, distinguishing between major and minor transcript variants [7]. Transcript levels correlate with disease burden and response to therapy, making PCR a reliable tool in over 95% of CML cases [8].

FISH, using labeled DNA probes, provides a highly sensitive and specific means of identifying chromosomal rearrangements such as the BCR::ABL1 fusion, even at low disease burden. It remains a valuable tool for detecting minimal residual disease and confirming Philadelphia chromosome-positive CML [9].

However, as demonstrated in this case, rare instances may arise where PCR results are negative despite FISH confirmation of BCR::ABL1. This discrepancy, observed in 2-4% of cases, can be attributed to atypical or cryptic BCR::ABL1 transcripts that are not detected by conventional PCR assays. These may result from unusual breakpoints or insertions that escape detection due to the assay’s primer specificity [10].

A negative PCR result in a clinically suspicious case of CML warrants further evaluation using complementary techniques such as FISH, next-generation sequencing (NGS), or cytogenetic analysis. Identifying the presence of BCR::ABL1 is critical for establishing the diagnosis and guiding treatment decisions, particularly the use of tyrosine kinase inhibitors.

## Conclusions

Myelofibrosis, a rare and aggressive subtype of myeloid malignancy, is marked by progressive bone marrow fibrosis and hematologic dysfunction. Although uncommon, it can evolve into chronic myeloid leukemia (CML), signaled by the emergence of the BCR::ABL1 fusion gene-the defining genetic hallmark of CML. Detection of this fusion gene is typically achieved through PCR, which identifies specific BCR::ABL1 transcript variants. However, in rare instances, PCR may yield false-negative results if the fusion occurs at atypical breakpoints not covered by the assay. In such cases, fluorescence in situ hybridization (FISH) proves invaluable, as it can detect the BCR::ABL1 gene rearrangement regardless of the exact breakpoint location. This highlights the importance of using complementary diagnostic techniques when evaluating suspected CML, especially in patients with unusual clinical presentations or atypical genetic profiles.
